# Analysis of the Cost and Efficacy of Intra-Articular Knee Injections

**DOI:** 10.5435/JAAOSGlobal-D-21-00203

**Published:** 2022-02-18

**Authors:** Sean Pirkle, Henry Seidel, Sarah Bhattacharjee, Lewis L. Shi, Michael J. Lee, Jason A. Strelzow

**Affiliations:** From the Department of Orthopaedics and Sports Medicine, University of Washington, Seattle, WA (Pirkle); the Pritzker School of Medicine, University of Chicago, Chicago, IL (Seidel, Bhattacharjee); and the Department of Orthopaedic Surgery and Rehabilitative Medicine, University of Chicago Medicine, Chicago, IL (Shi, Lee, and Strelzow).

## Abstract

**Introduction:**

Intra-articular joint injections have been used with the goal of providing patients with symptomatic relief. Recently, however, the efficacy of corticosteroid (CS) and hyaluronic acid (HA) injections in the management of knee osteoarthritis (OA) is questionable. In this analysis, we investigated the costs associated with injections by assessing overall use, conversion and average time to total knee arthroplasty (TKA), and reimbursement.

**Methods:**

Patients aged 50 to 70 years with a knee-related diagnosis of pain, effusion, or OA were identified in the Humana insurance national data set. Patients who received intra-articular injections were stratified by the type and number of injection(s) received. The subsequent rates of TKA were compared with Kaplan-Meier curves for patients who underwent CS injections, HA injections, and a benchmarking cohort of patients with OA and no history of knee injections in the medical record. Average time to TKA was determined from index diagnosis, and total cost was compared using Wilcoxon rank sum analyses.

**Results:**

A total of 778,686 patients were identified. Of these, 637,112 had no knee injection history, while 124,129 received CS and 17,445 received HA injections. The 10-year conversion to TKA was highest in HA cohort (31.6%), followed by the CS cohort (24.0%) and the noninjection cohort (7.3%) (*P* < 0.001). Time to TKA increased with number of injections for both injection types. For patients who underwent TKA, median cost was greater in HA ($16,687) and CS ($15,563) cohorts relative to noninjection cohort ($14,733) (*P* < 0.001).

**Discussion:**

Compared with the noninjection cohort, both HA and CS cohorts experienced increased costs and increased time to TKA. However, while the cost incurred in HA cohort was greater than that in CS cohort, no appreciable benefit was demonstrated for conversion or time to TKA. Therefore, if intra-articular knee injections are indicated for the nonsurgical management of knee OA, the results of this study support CS over HA.

Knee osteoarthritis (OA) is one of the most frequently encountered orthopaedic conditions, affecting more than 9.3 million adults and accounting for more than $27 billion in annual healthcare expenditures in the United States alone.^1,2^ Although OA is known to be caused by wear and tear on knee joint cartilage,^3^ current treatment modalities target different aspects of the condition and follow a tiered approach.^4-6^ Medical management often begins with acetaminophen and nonsteroidal anti-inflammatory drugs to address inflammatory mediators, while physical therapy improves outcomes for symptomatic pain and stiffness.^7^ As the condition severity worsens, affected joints may be managed with intra-articular injections or operative management with total knee arthroplasty (TKA).^8^

The efficacy of joint injections, however, presents a contentious topic. While corticosteroid (CS) injections have been repeatedly found to offer rapid onset pain relief, they have not demonstrated long-term effects.^9^ Although they are the most widely used intra-articular therapy for OA,^10^ reviews on CS injections have reported pain relief duration spans as short as 1 week^11^ and up to 6 weeks,^12^ while effects past that window remain under debate.^4,12^ CS injections may paradoxically induce cartilage damage over time and increase the risk of OA progression.^13^ Similarly, the outcomes of hyaluronic acid (HA) injections are contested, with the long-term efficacy of this treatment modality unproven. Comparatively, the pain relief provided by HA seems enduring than that by CS injections, with several studies suggesting HA outperforms CS beyond 8 weeks from treatment.^14^ HA is also designed to restore and protect the physiologic environment of the joint capsule, which, in principle, is a positive effect on function.^15^ However, perceived benefits are weighed against a higher price point and the context of industry-backed studies with a preponderance for many of the positive findings currently published in peer-reviewed journals.^16^

Notwithstanding injection type, outcomes studies published in the orthopaedic literature primarily focus on short-term pain scales to compare injection types.^4,9,11,12,14^ Because the injections themselves are designed with the goal of symptomatic relief in avoidance of escalated care in the form of joint replacement, using TKA as the foundation of a clinical analysis is justified if not warranted. With this study, we sought to investigate the trends related to the proportion of patients with OA receiving injections and to report the number of CS and HA injections received by patients and the efficacy of intra-articular joint injections regarding rate and time to TKA. Moreover, we aimed to examine the reimbursement related to various treatments for knee OA and the associated costs in all patients treated with intra-articular joint injections and perform a subanalysis of those who specifically went on to receive TKA.

## Methods

### Database

This study was conducted within PearlDiver, a national administrative claims database with more than 25 million patients. Records span from the years 2008 to 2018. With medical, prescription, and laboratory data, records are searchable through physician billing codes, including Current Procedural Terminology (CPT) and International Classification of Disease, 9th and 10th revision (ICD-9 and ICD-10) codes.

### Patient Selection

All patients who underwent an intra-articular knee injection were identified by CPT-20610. Because large joint injections may not be specific to the knee, a knee-related diagnosis code for knee pain, effusion, or OA was required to be present on the same day as the injection procedure. Patients were then stratified by the type of injection administered based on Healthcare Common Procedure Coding System J codes for either CS or HA injections. Laterality of the injection and the injection type were confirmed with CPT modifiers, and the number of injections administered within patients' record was determined using count codes with each instance of an injection counted as a single injection; a series of shots was counted as multiple shots in this study and not a single event. Patients lacking laterality modifiers or patients who received both injection types (CS and HA) were excluded. Any patient with a history of platelet-rich plasma injection into the knee joint was excluded as well. A comparator group was defined by patients with the knee-related diagnosis codes for knee pain, effusion, or OA who had no history of intra-articular knee injections at any point in their disease course. Finally, all patients were restricted to those between the ages of 50 and 70 years at the time of their first injection or knee-related diagnosis. The selected age range was chosen because it represents a time when patients are generally optimal candidates for both joint injection and TKA.

Because patients in PearlDiver are accessible through deidentified insurance records, data may not be reported secondary to a change of insurance, loss to follow-up, or death, for instance. To minimize the effect of reporting bias, when indicated, patients were excluded from further study when they were found to no longer have active records in the data set, with active records defined as uninterrupted enrollment with the insurance carrier.

### Outcome Measures

The rate of ipsilateral TKA and the average time to TKA from the date of indexed knee-related diagnosis was assessed for all patients. Patients were further stratified by type of injection and number of injections administered. A cost comparison was performed to determine any differences in reimbursement between injection cohorts and control subjects. Reimbursement data included all hospital and/or clinic visits, imaging, physical therapy, and prescription medication filled, provided the knee-related diagnosis was coded as either the primary or secondary problem in the patient record. The terms cost, payment, and reimbursement are used interchangeably and represent the actual amount paid by the insurer. The median reimbursement for the continuum of care received from initial knee-related diagnosis was first assessed for all patients in this study. Subsequently, a calculation of the median reimbursement for those who converted to TKA was conducted and stratified by treatment wing (noninjection, CS, and HA). For patients who were managed nonsurgically, cost collection stopped at the end of the study period; for patients treated surgically, tracking was halted the day after surgery.

### Statistical Analysis

All nominal variables were compared using chi-square tests. Conversion to TKA was evaluated for the injection cohorts and the noninjection group using Kaplan-Meier survival curves with log-rank tests. Statistical significance was set to *P* < 0.05. For nonnormally distributed data such as reimbursement, Wilcox rank-sum tests were used to compare cohorts with median, first quartile (Q1), and third quartile (Q3) used as descriptive statistics to minimize skew by outlying individuals.

## Results

### Rate of Intra-articular Injections

A total of 778,686 patients matched the inclusion criteria. Statistical differences in age, sex, race, and Elixhauser Comorbidity Index existed between the three treatment arms, with demographic information presented in Table 1. Among these patients, 637,112 did not have a history of intra-articular knee injections, while 141,574 received at least one intra-articular knee injection (86.7% CS versus 13.3% HA).

**Table 1:** Baseline Characteristics of Patient Cohort

	Patient Group			*P*-Value
	Noninjection	CS	HA	
	N = 637,112	N = 124,129	N = 17,445	
Characteristic				
Age				
50-54	96,761 (15.2)	15,112 (12.2)	1,967 (11.3)	<0.001
55-59	117,359 (18.4)	20,842 (16.8)	2,912 (16.7)	<0.001
60-64	128,501(20.2)	27,762 (22.4)	3,620 (20.8)	<0.001
65-69	294,491(46.2)	60,413 (48.7)	8,946 (51.3)	<0.001
Sex				
Female	382,330 (60.0)	79,709 (64.2)	11,021 (63.2)	<0.001
Male	254,782 (40.0)	44,420 (35.8)	6,424 (36.8)	
Race				
Asian	3,369 (0.5)	411 (0.3)	64 (0.4)	<0.001
Black	78,441 (12.3)	13,152 (10.6)	1,521 (8.7)	<0.001
Hispanic	7,980 (1.3)	1,071 (0.9)	130 (0.7)	<0.001
Native	1,077 (0.2)	164 (0.1)	31 (0.2)	0.012
White	331,129 (52.0)	67,868 (54.7)	9,982 (57.2)	<0.001
Unknown	215,116 (33.8)	41,463 (33.4)	5,717 (32.8)	0.002
ECI	5.83 ± 4.44	6.09 ± 4.40	5.78 ± 4.27	<0.001

ANOVA = analysis of variance, CS = corticosteroid, ECI = Elixhauser comorbidity index

Groups were compared using Chi-square analyses for categorical variables and ANOVA for linear variables.

*P* is significant at < 0.05.

Of the patients receiving CS injections, most (65.0%) were administered only one injection with fewer receiving 2 (21.6%), 3 (5.9%), 4 (3.5%), and 5+ (4.0%) injections (Table 2). Of the patients receiving HA injections, 25.6% were administered only one injection, with a comparatively larger proportion receiving multiple injections (2 (16.8%), 3 (25.0%), 4 (7.1%), and 5+ (25.5%)). In both injection cohorts, the gross number of injections was not found to be associated with conversion to TKA.

**Table 2:** Rate of TKA Stratified by Number of Injections Received

Patient Group	N Total	N TKA	Rate of TKA
Noninjection	637,112	20,140	0.0316
CS			
1 injection	80,664	6,906	0.0856
2 injections	26,826	2,161	0.0806
3 injections	7,322	790	0.1079
4 injections	4,377	347	0.0793
5+ injections	4,940	344	0.0696
HA			
1 injection	4,463	636	0.1425
2 injections	2,929	273	0.0932
3 injections	4,353	625	0.1436
4 injections	1,243	117	0.0941
5+ injections	4,457	349	0.0783

CS = corticosteroid, HA = Hyaluronic acid, TKA = total knee arthroplasty

Noninjection represents patients who did not receive intra-articular knee injection throughout the study period.

### Comparison of CS and HA Injections

**Figure 1:**
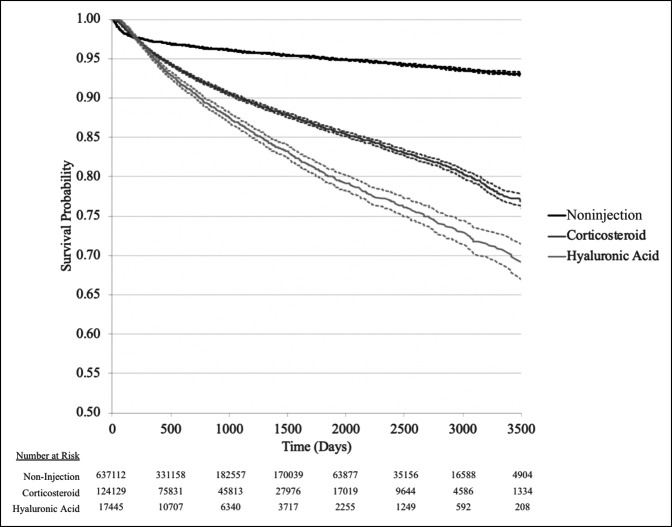
Graph showing Kaplan-Meier survival curve of rate of total knee arthroplasty in patients with a diagnosis of knee osteoarthritis, pain, or effusion. Noninjection represents patients who did not receive intra-articular knee injection throughout duration of study period; Corticosteroid represents patients receiving one or more intra-articular corticosteroid injections before total knee arthroplasty (TKA). Hyaluronic acid (HA) represents patients receiving one or more intra-articular hyaluronic acid injections before total knee arthroplasty. *P* < 0.001, log-rank analysis.

When directly comparing the two injection cohorts regarding the rate of TKA, CS injections were found to significantly outperform HA. After 1 year of knee-related diagnosis, 4.4% of patients treated with CS injections had undergone TKA. For patients active at 10 years follow-up, 24.0% had undergone TKA. The rates were 5.1% and 31.6% at 1 and 10 years for patients treated with HA injections, respectively (Figure 1; *P* < 0.001). For patients who did not receive an injection, 7.3% had undergone TKA by 10 years after diagnosis.

**Figure 2:**
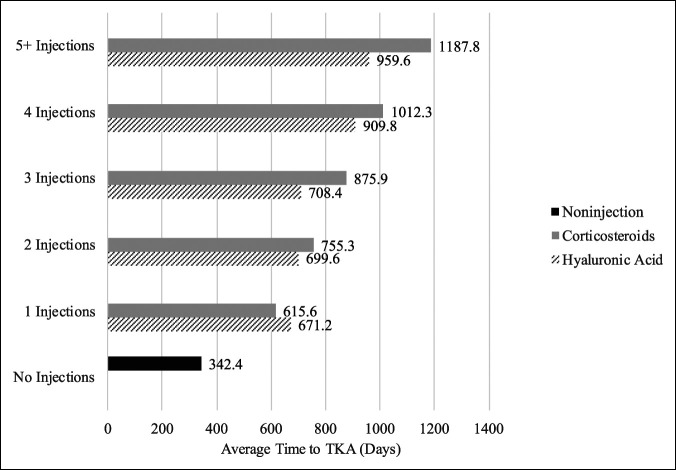
Graph showing average time to total knee arthroplasty (TKA) stratified by number of injections received and injection contents. Noninjection represents patients who did not receive intra-articular knee injection throughout the study period.

When investigating average time to TKA for the subset of patients receiving injections who ultimately converted to TKA, one CS injection was associated with an average time to TKA of 615.6 days; one HA injection was associated with a time to TKA of 671.2 days. Among patients receiving more than one injection, CS was associated with greater time to TKA relative to HA, with approximately 143.1 days gained with each additional injection and nearly double that of HA (average of 72.1 days gained per additional injection). For the noninjection group, the average time to TKA was observed to be 342.4 days from knee-related diagnosis. A comparison regarding time to TKA is fully depicted in Figure 2.

### Cost Analysis

**Figure 3:**
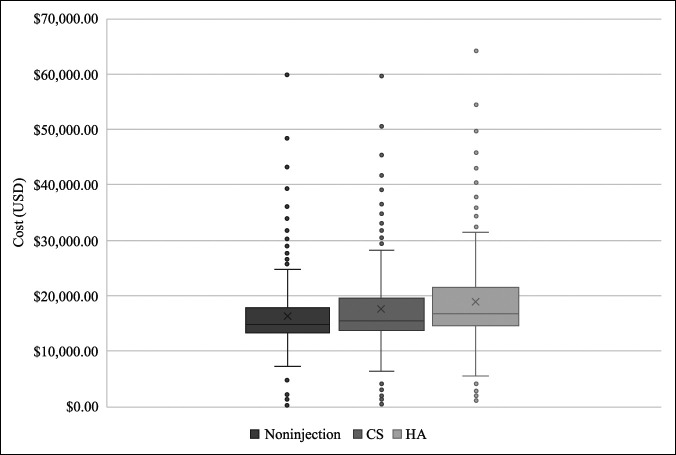
Graph showing total cost of care for patients diagnosed with knee osteoarthritis, pain, and effusion who required total knee arthroplasty (TKA) after initial treatment, stratified by treatment received. All prices are reported in US dollars (USD). Q1 = first quartile, Q3 = third quartile. *P* < 0.001, Wilcoxon rank-sum test.

In our analysis of cost, the median reimbursement for the care received for knee-related diagnoses without intra-articular injection administration was $178 (Table 3). The median cost was relatively higher in patients receiving intra-articular joint injections (CS: median cost $601 in patients receiving one injection and $1610 for five or more; HA: median cost $1554 in patients receiving one injection and $2752 for five or more). With an increasing number of injections received, whether CS or HA, the associated costs rose sequentially. For patients requiring TKA for the treatment of their knee-related diagnosis, the median cost was significantly greater in HA patients ($16,688) relative to CS ($15,563; *P* < 0.001) and the noninjection group ($14,733; *P* < 0.001) (Figure 3).

**Table 3:** Total Cost of Care for Patients Diagnosed With Knee Osteoarthritis, Pain, Effusion Stratified by Treatment Received, and Number of Injections (if Applicable)

	All Patients				TKA Patients			
	N	Q1	Median	Q3	N	Q1	Median	Q3
Noninjection	637112	71	178	611	20,140	13,205	14,773	17,854
CS								
1 injection	80,664	260	601	2194	6,906	13,728	15,408	19,141
2 injections	26,826	387	834	2,945	2,161	13,879	15,617	19,484
3 injections	7,322	564	1225	4,518	790	14,112	16,080	21,450
4 injections	4,377	621	1,250	3,782	347	14,026	16,076	20,639
5+ injections	4,940	848	1,610	4,226	344	13,973	16,418	22,278
HA								
1 injection	4,463	824	1,554	5,085	636	14,390	15,978	19,603
2 injections	2,929	932	1,733	4,140	273	14,161	16,624	21,758
3 injections	4,353	947	1,661	4,777	625	14,657	16,533	21,360
4 injections	1,243	1,326	2,270	4,935	117	15,125	17,557	22,791
5+ injections	4,457	1,612	2,753	5,834	349	15,371	17,498	23,541

CS = corticosteroid, HA = hyaluronic acid, Q1: first quartile; Q3: third quartile, TKA = total knee arthroplasty

All patients represent all patients matching inclusion criteria, regardless of treatment outcome. TKA patients represent a subset of total cohort who required TKA after initial treatment with or without intra-articular joint injection. All prices are reported in US dollars (USD).

## Discussion

TKA offers profound benefits to patients in both pain alleviation and functional outcomes.^17,18^ However, for some patients with knee OA, TKA may not be a viable treatment, and even among surgical candidates, many may seek to delay or avoid surgery altogether. A number of reasons for this have been cited, with the most frequently reported including concerns over interference with work and life-events and perception of surgery-related pain.^19,20^ For the latter group, intra-articular injections are a common strategy for providing symptomatic relief while potentially postponing the need for TKA because roughly 30% of TKA patients receive knee injections before their procedure.^21,22,23,24,25,26^

Despite their popularity, the use of injections to prevent disease progression has been driven more so by dogma than contemporary evidence-based literature because neither CS nor HA injections have been consistently shown to prevent conversion to or delay TKA. In fact, in the 2013 guidelines for the nonsurgical treatment of osteoarthritis, the AAOS issued a strong recommendation against the use of HA injections,^27^ finding that the current literature was unable to demonstrate a clinically effective response to HA injections.^6^ In addition, the guidelines could not recommend for or against the use of CS injections due to conflicting evidence.^28^ Because of this ambiguity, in this study we sought to define the clinical utility of intra-articular injections and compare which injection type carries the most value, if any at all.

In this study, 18.2% of patients with knee-related diagnoses underwent intra-articular injection. Although somewhat lower than rates reported in the literature, to isolate the effect of each type of injection, we excluded individuals fitting criteria for both treatment groups (and any platelet-rich plasma injection) and, consequently, effectively lowered the proportion of patients receiving injections. Of those included in either the CS or HA injection group, CS injections alone were far more common than HA injections alone. Of interest, an increasing number of joint injections received, hypothesized to be an indicator of severity of knee joint pathology, did not correlate with an increased likelihood of conversion to TKA in either injection cohort. The authors conjecture this is possibly due to an overrepresentation of patients who may not be surgical candidates and were thus more likely to receive strictly intra-articular injections as definitive management of their OA.

Overall, for patients receiving one injection who ultimately went on to TKA, both groups (CS, 615.6 days; HA, 671.2 days) experienced greater time from diagnosis to TKA compared with noninjection group (342.4 days). Therefore, we assume that the differences in the total number of days would suggest a delaying effect from the injection on the time to TKA. For each subsequent injection after the first, HA was found to offer less benefit when compared with CS (average + 143.1 days from diagnosis to TKA for the CS cohort and +72.1 days for the HA cohort). Of note, HA injections are often marketed to consumers in bundled packs (e.g., three and five). The number of patients observed receiving HA injections was consistent with this practice because most patients received one, three, or five injections total, with fewer receiving two or four injections. Although both groups were noted to have better outcomes relative to patients who did not receive injections regarding time from knee-related diagnosis to TKA, in contrast to industry claims,^9,16^ these data suggest decreasing marginal returns with increasing number of HA injections. These bundled packs are not specifically coded for billing purposes, so it cannot be confirmed at what stage of a bundled pack patients may be at or whether they are participating in a bundled treatment therapy at all, nor can the subjective experience of symptoms at time of HA injection be as specific as that of CS. Accordingly, the utility of comparing multiple HA injections with multiple CS injections is restricted by the bundled method of delivery.

When directly comparing conversion to TKA in CS group with that in HA group over the course of the 10-year follow up, CS (24.0% at 10 years) cohort was found to outperform HA cohort (31.6% at 10 years), although both cohorts were observed to fare worse than their noninjection counterpart (7.3% at 10 years). Furthermore, CS was found to be significantly more cost effective relative to HA. Although both injection modalities may initially result in lower costs for patients, over time, the utilization of injections for OA accrue increased downstream healthcare expenses in comparison with patients who elect to initially undergo TKA.^29^ The differences in cost observed in this study are similar to those in previous studies, which found the average cost of CS injections to be $437 in 2013^2^ compared with $900 per HA injection in 2013.^30^ Although HA was more expensive compared with CS, we did not observe a clinical benefit in this analysis.

## Limitations

With this study, we analyzed the records of nearly 800,000 knee OA patients and investigated the management strategies for their OA, the rate of conversion to TKA, and the reimbursement data linked to outcomes as measured by primary joint replacement. However, as with any large database study, we are limited by a number of factors. First, the accuracy of the data analyzed is dependent on physician billing codes from numerous providers and care settings. Second, by analyzing patients who only received either CS or HA injections, we cannot comment on the potential effects of receiving both types of injections. In addition, some degree of controversy exists regarding the specific molecular structure of the HA injected and its variable effect on disease progression,^31,32^ details that are not specified in the patient record. Because of this, all HA types are therefore treated as a single group. The issue of differences exists in baseline OA because our analysis begins at the first instance of a diagnosis code for knee OA, which may differ in severity between patients and cannot be confirmed with radiographic evidence. By aggregating thousands of OA patients in each of our cohorts and excluding patients with contralateral disease or multiple injection types received, we have attempted to minimize the degree of heterogeneity. Of course, when comparing patients who received injections to those who did not, it is likely the case that patients receiving injections are at different stages along the management paradigm for their disease and therefore have requested or been advised to undergo these treatment options. The survival curves plotting conversion to TKA reflect this assumption. Thus, although our rate of conversion in patients who did not undergo injections is consistent with published studies using similar methodologies,^33^ the noninjection cohort is primarily useful as a benchmarking group for providing context to the rates observed in the injection cohorts, rather than as a control group.

## Conclusion

In conclusion, we recommend counseling patients that neither CS nor HA injections were particularly successful in staving off eventual conversion to TKA. However, for patients seeking nonsurgical treatment, intra-articular joint injections—particularly CS injections over HA—may offer a short-term cost-effective option in the management of knee OA.
